# Cultural Adaptation and Psychometric Properties of the Persian Version of the Affordance in the Home Environment for Motor Development

**Published:** 2019

**Authors:** Somaye KAVOUSIPOR, Mehdi RASSAFIANI, Abbas EBADI, Farin SOLIMANI, Ali HOSSEINI, Carl GABBARD

**Affiliations:** 1 Occupational Therapy Department, University of Social Welfare & Rehabilitation Sciences, Tehran, Iran.; 2 Behavioural Sciences Research Centre (BSRC), Nursing Faculty, Baqiyatallah University of Medical Sciences, Tehran, Iran.; 3 Pediatric Neurorehabilitation Research Center, University of Social Welfare & Rehabilitation Sciences, Tehran, Iran.; 4 Department of Health & Kinesiology (Motor Neuroscience), Texas A&M University, Texas, United States

**Keywords:** Home environment, Assessment, Validity and reliability, Motor ability, Affordance

## Abstract

**Objectives:**

This study aimed to translate the Affordance in the Home Environment for Motor Development (AHEMD) scales (3-18 months (IS) and 18-42 month (SR) versions) into Persian and examine its cultural adaptation and psychometric properties.

**Materials & Methods:**

Four stages process was conducted as translation of the questionnaires (use of International Quality of Life Assessment protocol), cultural adaptation, and determination of its psychometric properties. Qualitative judgments were provided by 11 experts and 30 mothers for interview sessions. Quantitative data were gathered using 212 mothers.

**Results:**

For the IS version, validity values ranged from 0.63 to 0.95. *Intraclass* correlation *coefficient *for reliability of total score was 0.87 (0.83-0.97) and was Cronbach’s alpha (0.75). Validity for the AHEMD-SR ranged from 0.63 to 0.90, with reliability for total score of 0.98 (0.98-0.99, ICC). Cronbach’s alpha for this version was 0.84. Correlations with SES were significant for both scales: IS (*r* = 0.40) and AHEMD-SR (*r* = 0.42).

**Conclusion:**

Both translated versions of the AHEMD were valid and reliable assessments of the home environment of Iranian young children.

## Introduction

Child motor development is a complex process which results from the interaction between genetics, epigenetics, and the environment (1-3). The role of the home as the first environment for child experiences is very important for motor development, especially during infancy (4). Thus, a more supportive and stimulating environment is associated with more affordances and challenges for infant motor development that may lead to improvement in social and cognitive development (5). With regards to special populations, one out of six children has a developmental disorder caused by various factors, one of which can be environmental deprivation and lack of a stimulating home environment (6, 7).

An effective home environment includes educational and learning materials (e.g., toys, books), space, and stimulation from family members. All of which, have been shown to influence a child’s motor, cognitive and other behavioral aspects of development (8). From the Gibsonian viewpoint, an ecological approach, the environment is referred to as “resources and opportunities for action”. Children learn by interacting with their environment as well as events and incidences (9). What the environment provides and offers to animals or organisms is called an “affordance”. In other words, opportunities (e.g., toys, equipment, events, space) that provide a stimulating link between the environment and organism to create a meaningful behavior. Affordances include the type of space we move in, the cup we drink from, and ways that we can manipulate a toy or piece of equipment (10-12).

Regarding assessment of the home environment, the Home Observation for Measurement of the Environment (HOME) Inventory was developed to investigate demographic and socioeconomic status characteristics of families, and the relationship between the home environment, social, emotional, and cognitive development in children. The HOME was organized along six different dimensions including 1) responsivity, 2) acceptance, 3) organization, 4) learning materials, 5) involvement, and 6) variety. Although the dimension of “learning materials” and “organization” in the HOME to some extent can be related to motor development, this questionnaire was not designed to appropriately examine the relationship between motor affordances and home environment (13,14). The Affordance in the Home Environment for Motor Development-Self Report (AHEMD-SR) was created in 2005 for ages 18 to 42 months, and more recently a version for ages 3 to 18 months (AHEMD-Infant Scale (IS)) was validated for use. As of with writing, the original English versions have been translated into eight languages and reported as valid and reliable in countries such as China, Poland, Lebanon, and Italy (15-17).

The population of children 4 yr old in Iran is estimated to be 3.5 million (18). Of that population, approximately 10% have developmental disorders (19). Fine- and gross motor skills, compared to US norms, were significantly lower in Iranian children under 4 yr of age. In those reports, one possible explanation for the differences was lack of motor affordances in the home (15, 20). However, there is little of any empirical evidence to support such an assumption. 

We aimed to modify the AHEMD (both versions) for using with Iranian children. Our objectives were: translate to Persian and examine its cultural adaptation and psychometric properties. 

## Materials & Methods

Creation of the Persian versions of the AHEMD was conducted in the following four phases. 

***Phase 1. Translating the questionnaires***

To translate the two questionnaires, we used the International Quality of Life Assessment (IQOLA) protocol (21). First, the original versions were translated from English to Persian by two translators, who were also experts in activities related to children (one was also an occupational therapist). After completion of the forward translation, both Persian translations were merged and a single translation for each questionnaire was created. Then, the Persian translations were back-translated into English by two other experts in English translation. Finally, both back-translated versions were merged and a single back-translated for both questionnaires was obtained through a meeting with translators and research team members. These finalized questionnaires were compared with the original versions and discussed by a panel of experts until a consensus was achieved. Then, the questionnaires were emailed to the original authors who confirmed them as acceptable. 

***Phase 2. Investigating cultural adaptation***

For cultural adaptation considerations, an expert panel was held with the presence of six occupational therapists, two experts in developmental medicine, two speech therapists, one ergonomist, and one environmental psychologist. Together these experts had an average of 15.54 ± 3.49 yr of experience in their fields of expertise. Each sentence was reviewed by panel members in consideration of fluency of sentences and Persian grammar/expressions. With consensus, sentences and expressions were altered. Sentences with “first-person pronouns” were changed to third person pronouns for clearer understanding in Persian. For example, the sentence *(I / We usually has a daily time for playing (interacting) with our child) was changed to Do you and your spouse spend some time for playing with your child during a day?*

In addition, the main structure of the questionnaires which was in the form of a table with separate rows in such a way that lines related to columns were faded, was altered into a table with separate columns and rows to make each of the three subtests more visible. In the original questionnaires, instruction for how to complete the questionnaire was provided only at the beginning of the 3 to 18-month questionnaire (IS), not with the version for older children (SR). The expert panel decided to add the instruction part at the beginning of AHEMD-SR too. Moreover, in the “help” section of the questionnaires, some explanation about the concept of ‘inside space’ and ‘outside space’ was provided. In addition, the picture of some toys was not tangible for typical Iranian children; thus, by searching toy shops and websites of reputable toy stores, some pictures of toys were changed to make it familiar for the Iranian population. With this change, the similarities of the toys were considered.

***Phase 3. Investigating the content and face validity***

To investigate content validity, questionnaires were given to 11 child development experts who participated in the cultural adaptation phase. Then content validity ratio (CVR) was used to examine relevancy, clarity, and simplicity. Individualized-Content Validity Index (I-CVI) was used to investigate relevancy of questions and the Scale-Content Validity Index (S-CVI) for the total score of questionnaires (18). For evaluating face validity, questionnaires were given to 15 mothers who had 3 to 18 months old (mean age was 28.86 yrs., 8 participants had high school diploma and lower, and 7 had attained a higher education level), and 15 mothers who had children 18 to 42 months of age (mean age 29.33 yrs. old, 7 participants had a high school diploma and lower, and 8 had higher education diplomas). A diverse range of SES describes both groups. Through person to person and group interviews, participants were asked to state their understanding of instructions and each question. Accordingly, at this stage, some parts of the questionnaires were modified based on the participants’ feedback.

***Phase 4. Assessing internal consistency, test-retest reliability and convergent validity with Social Economic Status (SES)***

The questionnaires fluency and understandability were validated and adapted for the Iranian culture based on experts and mother opinion. Overall, 62 mothers of children 3 to 11 months of age and 50 mothers with children12-to 18 months of age (mean of 9.92 months +3.99 SD), and 100 mothers with children 18 to 42 months of age (mean 28.41+7.2 months) were randomly selected from three main health and vaccination centers in Shiraz, Iran. Call numbers and required healthcare information were obtained through health care registry. Mothers with typically developing children with no physical, psychological, or significant cognitive disorders were selected and invited to participate. Both versions of the AHEMD were completed again by 83 mothers (46 mothers with children 3-to 18 months and 37 with children 18 to 42 months); the remainder did not participant in the Retest phase (Table 1). 

***Data collection***

Information collected included the AHEMD-IS and AHEMD-SR, family background and family characteristics related to SES. For test-retest reliability, questionnaires were retaken by the original respondents after two weeks. 

***Measures***

 AHEMD-IS. The original English version of this instrument has been reported as a valid and reliable self-report questionnaire for evaluating the home environment by parents in children 3 to 18 months (19). Scoring information is available for two age’s groups: groups of 3 to 11 months and 11 to 18 months. There are four subtests including; Physical Space (PS) in Inside Space and Outside Space parts, Variety of Stimulation(VS), Fine Motor Toys(FMT), and Gross Motor Toys (GMT) used to assess the home as ‘excellent’, ‘adequate’, ‘moderate adequate’ and ‘less than adequate’. 

***AHEDM-SR***

This instrument is a valid and reliable questionnaire used to assess home affordance for children 18 to 42 months and has 5 subtests including; Inside Space (IS), Outside Space (OS), Variety of Stimulation (VS), Fine Motor Toys (FMT) and Gross Motor Toys (GMT) (15). Categorization for this instrument is ‘good’, ‘moderate,’ and ‘poor.’ 

Additional information for both instruments and reported translation may be found at http://www.ese.ipvc.pt/dmh/AHEMD/ahemd_1.htm

***Social Economic Status (SES)***

Use of some properties such as parental education level, and family income can be used to evaluate SES (4-5), thus we combined some family characteristics including; mother education, father education, owning a house, family income, and house measurements, together to evaluate *SES. *Then, four groups of SES were created based on of percentiles of 25%, 50%, 75% and 100%.

**Table1:** Participant’s characteristics

	3-11month(n=62)	12-18momth(n=50)	18-42month(n=100)
Age(month)	6.93±2.26	13.42±2.76	28.41±7.2
Sex	51.6%female 48.4%male	46%female 54%male	40%male 60%female
Birth weight	3166.85±385.36(g)	3103.46±627.16(g)	3132.42±512.17(g)
Mother education	48.4%≤12 years 51.6%≤12 years	54%≤12 years 46%≤12 years	52%≤12 years 48%≤12 years
Mother age	28.74±4.99	31.08±4.89	30.23±5
SES	Class1 (37%) Class2 (31%) Class3 (20.1%) Class4 (12.9%)	Class1(18.2) Class2(52.4) Class3(15.7) Class4(13.7)	Class1(34.7) Class2(48.5) Class3(11.3) Class4(6.4)

SES: Social Economic Status

**Table 2:** Psychometrics properties of AHEMD-IS & AHEMD-SR

AHEMD-IS(3-18m)						AHEMD-SR(18-42m)			
		ICC (n=46) (CI95%)	IC (n=46)	Convergent with SES (n=112)	ANOVA	ICC (n=37) (CI95%)	IC (n=37)	Convergent with SES (n=100)	ANOVA
PS (OS&IS)		.96 (.93-.98)	.79	.31*	.004	.99 (.98-.99)	.97	.40*	.02
						.95 (.91-.97)	.79	.001	.26
VS		.93 (.88-.96)	.61	-.24	.1	.99 (.98-.99)	.64	.25	.91
GMT	≤11m	.78 (.51-.9)	.61	.42*	.002	.97 (.95-.99)	.71	.36*	.003
	>12m	.93 (.83-.97)	.66						
FMT	≤11m	.84 (.6-.93)	.65	.27*	.009	.96 (.94-.98)	.82	.27	.001
	>12m	.96(.84-.99)	.78						
Total score		.87 (.83-.97)	.75	.40*	.006	.98 (.98-.99)	.84	.42*	.00

ICC(Intraclass Correlation Coefficient),CI(Confidence Interval), IC(internal consistency), SES(social economic status),PS(physical Space),OS(outside Space), IS(internal Space),VS(variety of Stimulation), GMT( gross Motor Toys), FMT(Fine Motor Toys),FMT and GMT divided two group age(≤11m AND>12m_) in AHEMD-IS , ANOVA(Compare AHEMD in 4 groups of SES).

* correlation is significant at the .05 level

**Table 3:** Mean scores of affordance in the Iranian home (3-18, 18-42 month old age)

AHEMD subtest	AHEMD-IS			AHEMD-SR (n=100)		(American population)	Iran Compare with America
	Percentile of Total score categories (3-18m) (n=112)	11-18m (n=50)	3-11m (n=62)				
		Mean±SD (ranking)	Mean±SD (ranking)	Ranges	Mean±SD	Mean±SD	One sample t-test
PS (OS&IS)	Group(1): 35.7 % Group(2): 34.8% Group(3):21.4% Group(4): 8%	4.3±2.22 (3)	4.33±1.91 (2)	0-12	(OS) 3.88±2.05	2.1±1.9	T (6.95) p-value.00
				1-10	(IS) 6.85±1.96	8±1.6	T (-5.86) p-value(.00)
VS		14.36±1.8 (2)	11.82±3.09 (3)	14-29	23.9±3.69	12.2±1.8	T(29.16) p-value(.00)
GMT		8.52±2.89 (3)	6.58±2.25 (2)	4-45	20.24±8.82	21.4±10.4	T(.003) p-value.99
FMT		9.92±4.55 (3)	3.87±2.41 (3)	6-70	25.51±12.63	51.2±20.4	T (-20.33) p-value( .00)
total score		37±7.74 (2)	26.61±5.9 (2)	7.18-16.86	11.43±2.01	12.5±3.5	T (-5.09) p-value( .00)

(1): excellent, (2): adequate, (3): Moderately adequate, (4): less adequate. PS (physical Space),OS(outside Space), IS(internal Space),VS(variety of Stimulation), GMT( gross Motor Toys), FMT(Fine Motor Toys)

***Statistical analysis***

SPSS 21 (Chicago, IL, USA) was used to determine correlations between scores obtained from the initial testing and retests via *Intraclass *Correlation *Coefficient (ICC)*. Internal consistency was calculated using the Kuder–Richardson Formula 21(KR21) for yes/no questions and Cronbach’s α for Likert questions; a value more than 0.6 was accepted as adequate internal consistency (18). For Convergent validity, the relationship between each questionnaire and SES were evaluated using Pearson’s and Spearman’s rank correlation method. Significant level was set at 0.05. 

***Ethical consideration***

**Informed consent **was obtained from all parents participated in the project. Ethics Committee of the university approved the study as number # 912502005.

## Results

***Participants***

Participant number (noted earlier) varied according to the different phases of the study (Table 1). 

***Cultural adaptation***

Some structural modification was done for cultural adaptation. In addition, we changed the pictures of some questions related to FMT and GMT according to the expert panel’s feedback. Pictures of toys were changed in questions 20, 26, 28, and 30 in the AHEMD-IS and questions 45, 49, 51,52,55,58 in the AHEMD-SR.CVR and CVI were calculated to evaluate content validity for both questionnaires. For the AHEMD-IS, CVR was 0.63–1; I-CVI was 0.9-1, and S-CVI was 0.95. For the AHEMD-SR, CVR was of 0.63 -1; I-CVI was 0.81-1, and S-CVI was 0.9.

***Convergent validity of AHEMD-IS and AHEMD-SR***

The correlations between total AHEMD-IS and total AHEMD-SR were 0.4 and 0.42 (*Ρ*≤0.005). Significant correlations were found between SES and some subtests of the AHEMD-IS, including GMT, PS and FMT. The correlation between SES and VS was not significant (Table 2). For the AHEMD-SR, the only significant correlations were between SES and IS and SES and GMT. ANOVA results showed that total scores of both the AHEMD-IS and AHEMD-SR were significantly different between the four SES groups (Table 2). 

Reliability of AHEMD-IS and AHEMD-SR. Internal consistency of all subtests of both questionnaires ranged between 0.61-0.97. Results of the ICC showed high test-retest reliability (Table 2). 

***Descriptive statistics***

AHEMD-SR mean distribution was 13.43±2.01 (SD). Since this total score distribution showed a normal configuration, a Mean ± 1 SD interval was used to categorize scores into Low (less than 9.42 (Mean-SD), Average (9.42 to 15.44) and High (more than 15.44). AHEMD-IS mean distribution families with children ages 3 to 11 months was 26.61±5.9 (SD), and 37±7.74 (SD) for the families with children ages 11 to 18 months. The home environment for Iranian children ages 3 to 18 months, categorized level 2, would appear to be have provided ‘Adequate’ home motor affordances (Table 3).

Results for subtests of the AHEMD-IS showed that more than 50% of homes did not have adequate availability of gross- and fine-motor toys. Included were toys mentioned in questions 20, 24, 25, 28, 30, and 35 (materials that stimulate forms of locomotion such as rolling, creeping, crawling, roly-poly, pop-up, spinning toys, multi-activity tables, shape sorters, and puzzles (2–6 pieces). Deficiencies (more than 50% of homes lacking) for the AHEMD-SR included toys mentioned in questions number 42, 45, 48, 49, 50, 51, 52, and 54 (puppets, familiar play scenes, Lacing cubes or boards and large colored beads, pegboards, simple matching toys, pop-up-toys and jack-in-the-box toys, multi-activities tables, and large plastic bricks). 

## Discussion

Our results indicate that both translations of the AHEMD (IS and SR) are valid and reliable instruments for assessing the homes of Iranian children. The AHEMD-IS enjoys an acceptable internal consistency coefficient ranging from 0.61 to 0.78 in terms of all subtests; therefore, may be used for the section of the Physical Space with confidence, but in sections of VOS, GMT, and FMT, it should be employed with more caution. The ICC for different subtests of this questionnaire in the original research in USA ranged from 0.63 to 0.83. The internal consistency coefficients of the total score in the present study were 0.75, and in the original research in the USA was 0.83 (19).

The elimination of items 8, 9 and 14 in the present translation of AHEMD-IS increased Cronbach’s α similar to the original version (19). “Does the child play with other children on a regular basis?” “Do you regularly play with your child so that he/she learns about his/her body parts?” Most of the participants answered yes. These two items as well as the item “In tummy time play?” like in the original, had a ceiling effect. 

In AHEMD-IS, subtest of GMT that considered for ages 3 to 11 months, eliminating the item of blocks and cubes (plastic, sponge, cloth, cardboard, wooden, rubber) resulted in an inconsiderable increased Cronbach’s α. However, the research team decided to remain the item in its original format. 

Regarding ICC results and reliability, results indicated that both questionnaires have high internal consistency. The internal consistency for various subtests in this study was within the range of 0.78 - 0.96. In the original study, the final score was 0.94 (16). A comparison of results shows that our translations have acceptable psychometric properties. In order to investigate the convergent validity, the relationship between the questionnaires and the family’s SES was used and the findings indicated a significant relationship between SES and PS, GMT, FMT subtests as well as total score. Studies conducting to survey the relationship between SES and home environment showed similar results (22, 23).

 Concerning the relationship between the VS section and SES, while the relationship was not statistically significant and the item’s scores for different SES did not indicate any significant differences, the two had a reverse relationship; i.e., it appears that the higher the family status, the less interaction they had with their children. This can be explained by in higher-status families, parents limit their parental duties primarily to only buying toys and spending money on their children. Indeed, in order to verify this claim, a larger sample and more in-depth examination are required. Items for the home’s physical space and fine- and gross-motor toys in the AHEMD-IS questionnaire showed significant differences between the four SES groups. Thus, the higher a family’s SES in terms of education, income and welfare, the higher the likelihood of providing adequate motor affordances in the home (22). 

Interestingly, when comparing AHEMD-SR scores for Iranian and American samples, scores for OS and VS subtest, were significantly higher for Iranian children. This difference may be explained in part by something noted in a study using this instrument and conducted in China (15, 19). Local parks were frequently used by the child as the outside space. A finding that was also noted in our study. Speculatively, this interaction resolved many of the shortcomings in the home environment. In the VOS section, Iranian families spend more time interacting with their children. However, no significant relationship was observed regarding gross- and fine-motor toys in the home. Overall, Iranian children had lower scores than American children and this difference was significant (16, 17). Regarding the mean scores and home environment ranking among 3 to 18 months old children, about 30% of Iranian families had ‘relatively adequate’ and ‘less than adequate’ status. This percentage comprises a considerable proportion of the population and finding strategies to improve this situation essentially. 

**In conclusion, **both translated versions of the AHEMD, are valid and reliable assessments of the home environment of Iranian young children. Both questionnaires appropriate for research and clinical goals in Persian population.
